# MIMO radar DOA element position error correction method based on overlapping reference element matrix reconstruction

**DOI:** 10.1038/s41598-025-03276-1

**Published:** 2025-05-27

**Authors:** Feng Tian, Tianyu Wei, Weibo Fu, Siyuan Wang

**Affiliations:** 1 Communication and Information Technology, Xi’an University of Science and Technology, 710054 Xi’an, China; 2 Xi’an Key Laboratory of Network Convergence Communication, 710054 Xi’an, China

**Keywords:** Time Division Multiplexing Multi-Input-Multiple-Output (TDM-MIMO) radar, Overlapping array elements, Array element position error, Toeplitz matrix, Engineering, Electrical and electronic engineering

## Abstract

Aiming at the problem of the degradation of angle estimation performance of the MUSIC algorithm due to the position error of the array element, this paper proposes a MIMO radar DOA array element position error correction method based on overlapping reference array element matrix reconstruction. This method uses overlapping virtual array elements as references, corrects the array element position error by phase difference, and constructs an error compensation matrix to eliminate the virtual array position error. At the same time, the nuclear norm optimization is introduced to reconstruct the Toeplitz structure to reduce the influence of system noise and array element disturbance on angle estimation, and the MUSIC algorithm is used to achieve accurate angle estimation. Simulation results show that the angle estimation error of this method is 0.2$^{\circ }$ under a signal-to-noise ratio of 20 dB. The actual traffic scene verification shows that this method effectively improves the angle resolution capability of the MUSIC algorithm and meets the requirements of radar accuracy for traffic applications.

## Introduction

The road traffic environment is becoming increasingly complex, and the requirements for urban traffic systems are also increasing accordingly. Traffic radar^1^, as the main monitoring means for urban traffic, needs more accurate and efficient monitoring performance to meet the needs of urban traffic. Time Division Multiplexing Multi-Input-Multiple-Output (TDM-MIMO) system^2^ millimeter-wave(mmW) radar is widely used in traffic monitoring scenarios due to its advantages of high resolution, strong anti-interference and low cost. However, in the process of practical application, due to the limitations of antenna assembly and engineering manufacturing level, thus introducing the array element position error^3^, which affects the radar array vector and leads to the reduction of radar direction measurement accuracy^4,5^. Therefore, conducting research on array element position error correction methods is of great significance to improving radar direction finding accuracy.

In order to solve the problem of array error correction, array error correction mainly adopts discrete measurement, interpolation method and storage method. Most of these traditional methods rely on pre-calculation or storage of array manifold matrix, which not only increases the complexity of the measurement process, but also significantly increases the implementation cost and time overhead, and the correction accuracy also has certain limitations. With the continuous deepening of research on array error correction technology, related methods have been gradually improved. According to whether it relies on external auxiliary signal sources, the current mainstream error correction algorithms can be divided into two categories: one is the active correction method with the help of known signal sources, and the other is the self-correction method without additional calibration sources.

Active correction is to use an external correction source to correct the array. In early studies, scholars introduced a calibration signal source with a known position and used the maximum likelihood estimation algorithm to model and estimate the array errors caused by factors such as phase error, amplitude inconsistency, mutual coupling effect, and array element position deviation, thereby improving the direction finding accuracy and resolution to a certain extent. However, in practical applications, the computational complexity of the method using a correction source with a known position is relatively high. In response to this challenge, Bechter J et al.^6^ proposed a method of improving discrete Fourier transform (DFT) to compensate for the array element phase error without relying on virtual array elements, simplifying the algorithm implementation and effectively improving the stability of angle estimation. Dai Z et al.^7^proposed a joint gain-phase error correction method by introducing two independent calibration sources and utilizing the MUSIC for offline estimation. Lin Y et al.^8^ extracted the phase difference introduced by the target motion by processing the received signals of the reference array elements, and then compensated the array for the motion. Although the active correction method can effectively compensate for array errors to a certain extent, in practical applications, due to the difficulty in obtaining external calibration sources and the complexity of deployment, its engineering implementation has certain limitations and it is difficult to meet practical needs in complex environments.

In order to make up for the shortcomings of active correction methods, researchers use self-correction methods to correct array errors. Self-correction methods can be divided into two categories: one is based on the least squares criterion, by constructing an iterative function, cyclically optimizing the array error parameters and the direction of the incoming wave until the function converges to the optimal solution, thereby obtaining the corresponding azimuth and error estimation results; the other is based on maximum likelihood estimation or subspace fitting theory, constructing a likelihood function and performing a joint multi-dimensional search for the target direction and error parameters. Häfner S, Lee H et al.^9,10^ used the maximum likelihood estimation compensation method to address the phase error and Doppler ambiguity problems caused by motion. They reduced the error while improving the angle estimation accuracy. However, errors still exist at high signal-to-noise ratios, and MLE requires multi-dimensional non-convex optimization, which increases the amount of calculation and is not suitable for real-time systems or resource-constrained applications. Tian F, Baral A B et al.^11,12^ adjusted the MIMO array arrangement to form overlapping array elements, used the phase difference of overlapping array elements, and combined the iteration and construction cost function methods to achieve array element phase error compensation. However, the problem of outliers in computational complexity and low signal-to-noise ratio environments remains a significant challenge. Zhang X et al.^13^ performed singular value decomposition on the radar snapshots and transformed the phase error problem into a least squares problem to achieve array phase error correction. However, the estimation accuracy of this method depends on the amount of snapshot data. When the amount of data is small, the estimation result may be inaccurate. Maślikowski Ł et al.^14^ used two calibration methods, normalization and average value, and singular value decomposition, to calibrate the phase offset of the antenna array, but the accuracy was low under low signal-to-noise ratio. Zhao Z et al.^15^ proposed a method for error estimation and compensation of broadband radar arrays under multi-target conditions. They used the solution (LS) to estimate the inter-channel error and then combined it with a genetic algorithm to correct the array position error. However, the introduction of the genetic algorithm greatly increased the amount of calculation. Hu CN et al.^16^ proposed a self-correction algorithm for millimeter-wave MIMO array errors and applied genetic algorithms to large-scale MIMO arrays to improve the calibration efficiency. However, due to the large amount of computation required by genetic algorithms, their application to large-scale MIMO arrays cannot meet the real-time requirements of the system. Barthelme A et al.^17^ applied machine learning technology to array signal processing and used a neural network to extract samples from sub-arrays to reconstruct the entire covariance matrix. They achieved high estimation accuracy even under small sample conditions. The neural network method relies on a large number of training samples. In actual traffic scenarios, if the training data does not completely match the test environment, the generalization ability and robustness of the neural network method will decrease.

Due to the specific Toeplitz property of the covariance matrix in the MUSIC angle estimation algorithm, many scholars have done a lot of research around this feature. Yang J et al.^18^ proposed an iterative Toeplitz algorithm, which corrects the array position error by performing Toeplitz preprocessing on the covariance matrix of the received data, and combines it with the eigenvalue reconstruction method for joint iterative calculation to improve the angular resolution of multi-target signals. However, the iterative processing greatly increases the amount of calculation. Liu J et al.^19^ combined the gain phase error matrix estimation and the fast smoothed sparse signal reconstruction method to eliminate the problem of angle estimation degradation caused by gain phase error in the partially calibrated array. However, it relies on the information of some calibrated sensors, especially when the signal noise is large or the sensor calibration information is insufficient, which has certain limitations. Aoun Allah N. et al.^20^ used two Toeplitz matrix reconstruction methods to deal with the angle estimation problem in the coherent source scenario. They constructed a Toeplitz matrix, used the new Toeplitz matrix for eigenvalue decomposition, and calculated the cross-correlation vector between the first array element and the received signals of other array elements. Based on this, they constructed a Toeplitz matrix to remove the coherence between the signals, thereby improving the angle estimation accuracy. Wagner M et al.^21^ introduced the concepts of Vanderdoes matrices and irregular Toeplitz matrices to solve the DOA estimation method for non-uniform linear arrays and extended it to the Root-MUSIC algorithm to enable it to be used in the case of irregular sampling for efficient DOA estimation. Qi B et al.^22^ combined the Toeplitz matrix reconstruction method with quadratic spatial smoothing processing to improve the DOA estimation accuracy at low signal-to-noise ratio.

In summary, the research on array error correction has been continuously invested and deeply explored by many researchers at home and abroad. Although existing methods have made some progress in alleviating array element position errors, signal processing faces higher complexity due to the presence of a large number of moving targets in complex traffic environments. In addition, the inevitable noise interference and array structure disturbances in actual system operation also affect the accuracy of angle estimation, resulting in certain limitations of radar in high-precision traffic detection tasks. Therefore, improving the accuracy of multi-target angle estimation is of great significance to enhancing the practicality and reliability of millimeter-wave radar in the field of traffic monitoring.

To address the existing problems, we use the MIMO radar receiving and transmitting characteristics to form overlapping virtual array elements by reconstructing the antenna array arrangement, and realize the element position error correction based on the reference element compensation method; at the same time, we use the Toeplitz property of the array covariance matrix and adopt the matrix reconstruction method to reduce the influence of system noise and array element disturbance on angle estimation. Finally, we use the MUSIC algorithm to perform angle estimation, realize the element position error correction, and improve the angle estimation accuracy of the target vehicle.

## MIMO array signal modeling

According to the principle of MIMO virtual array extension, the formation of overlapping virtual elements must satisfy the following conditions:

1. The transmitting array is a uniform linear array.
2. The receiving array consists of two uniform linear arrays, and the total number of antennas is even.
3. The number of overlapping arrays is less than half of the number of receiving arrays.

**Fig. 1:**
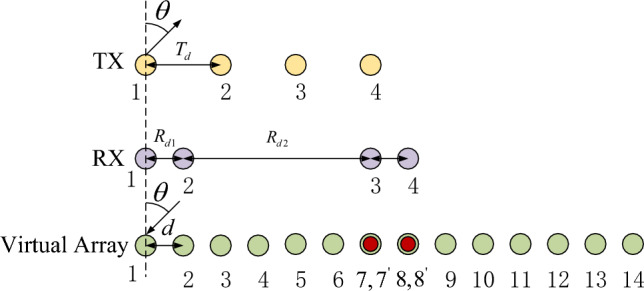
Overlapping MIMO antenna array (4TX-4RX).

We assume a transmitting array with *M* elements, arranged as a uniform linear array with element spacing $d_t = dN/2$. The receiving array has *N* elements, split into left and right sides: elements $1 \sim N/2$ on the left and $N/2 + 1 \sim N$ on the right. Both sides have element spacing $d_1 = d$, while the spacing between receiving array elements at *N*/2 and $N/2 + 1$ is $d_2 = [(M-1)N/2 - 1]d$.Each antenna element is isotropic, unaffected by channel inconsistency or mutual coupling. Using a virtual array expansion, an equivalent virtual receiving array is formed as a uniform linear array with *N* virtual elements and spacing $d = \lambda /2$ (where $\lambda$ is the wavelength). For example, in a 4-transmit, 4-receive MIMO array, the equivalent virtual receiving array configuration is shown in Fig. 1.The equivalent virtual array includes two pairs of overlapping array elements. In the first pair, the 1st transmitting array element transmits to the $(N/2 + 1)$th receiving array element, and the $M$th transmitting array element transmits to the $(N/2 - 1)$th receiving array element. This forms the first overlapping pair.In the second pair, the 1st transmitting array element transmits to the $(N/2 + 2)$th receiving array element, while the $M$th transmitting array element transmits to the $(N/2 - 2)$th receiving array element. These overlapping pairs have coincident phase centers.The MIMO array’s transmit antennas operate sequentially as TX1, TXM, TX2, ..., TXM-1. The period from the start of TX1 to the end of TXM-1 transmission constitutes one MIMO cycle^23^.

According to the TDM transmission method, the sawtooth wave transmission signal $S_T$ in the $k$th frequency modulation (FM) cycle is expressed as:

1 $$\begin{aligned} S_T(t) = A \exp \left\{ j 2 \pi f_0 \left( t - (k - 1) T \right) + \frac{\mu }{2} \left( t - (k - 1) T \right) ^2 + \varphi _0 \right\} + n(t), \;\;\;\;\;\;\;\;t \in \left[ (k - 1) T, \, T + (k - 1) T \right] \end{aligned}$$

where $A$ is the amplitude of the transmitted signal, $f_0$ is the initial frequency, $\varphi _0$ is the initial phase, $n(t)$ is the noise contained in the echo signal, and $\mu$ is the FM slope expressed as:

2 $$\begin{aligned} \mu = \frac{B}{T} \end{aligned}$$

where $T$ is the linear FM modulation period and $B$ is the FM bandwidth.

In order to obtain the target distance, speed, and angle information, it is necessary to extract the echo signal. The echo signal is mixed with the transmission signal and filtered through a low-pass filter to obtain an intermediate frequency signal. The intermediate frequency signal received by the $n$-th virtual array element is expressed as:

3 $$\begin{aligned} S_{IF}(t)&= S_T(t) \times S_T^*(t) \nonumber \\&\approx A_n \exp \left[ j 2 \pi \left( \mu \Delta t_r t + f_0 \Delta t_v \right) \right] \exp \left[ j 2 \pi f_0 \Delta f_k \right] + n(t) \end{aligned}$$

where $A_n$ represents the amplitude of the echo signal, $\Delta t_r$ is the time delay caused by the radial distance of the target relative to the radar, $\Delta t_v$ is the time delay caused by the radial speed of the target relative to the radar, and $\Delta t_{\text {ant}}$ is the time delay caused by the antenna array spacing.

The mixed signal is low-pass filtered to remove the high-frequency signal. The simplified intermediate frequency signal can be expressed as:

4 $$\begin{aligned} S_{IF}(t, n) = S_{IF}(t) \exp \left[ -j \varphi _n \right] + n(t), \end{aligned}$$

where $\varphi _n$ denotes the phase information of the $n$-th virtual array element.

We use a 4-transmit 4-receive antenna array, that is $M = 4, N = 4$. Assuming that the signal amplitude of each cycle is equal, $\varphi _q$ of the $q$-th virtual array element is expressed as:

5 $$\begin{aligned} \varphi _q = {\left\{ \begin{array}{ll} (q-1)\varphi _d, & q = 1, 2, 7, 8 \\ (q-1)\varphi _d + \varphi _v, & q = 7', 8', 13, 14 \\ (q-1)\varphi _d + 2\varphi _v, & q = 3, 4, 9, 10 \\ (q-1)\varphi _d + 3\varphi _v, & q = 5, 6, 11, 12 \end{array}\right. } \end{aligned}$$

where $\varphi _q = 2 \pi f_0 \Delta t_{\text {ant}} = 2 \pi d \sin \theta / \lambda$ represents the phase difference corresponding to the propagation path of electromagnetic waves between adjacent array elements; $\varphi _r = 2 \pi f_d T$ represents the phase difference introduced by the target motion between adjacent array elements, and $f_d = 2v / \lambda$ is the Doppler frequency of the target motion.

In Eq. (5), overlapping array elements are formed at the positions of the 7th and 8th array elements, and the total number of virtual array elements is $Q = M \times N - 2$.

## MIMO radar angle estimation error analysis

In this section, we analyze the effect of array position error on angle estimation, including its impact on the array phase and covariance matrix.

**Fig. 2:**
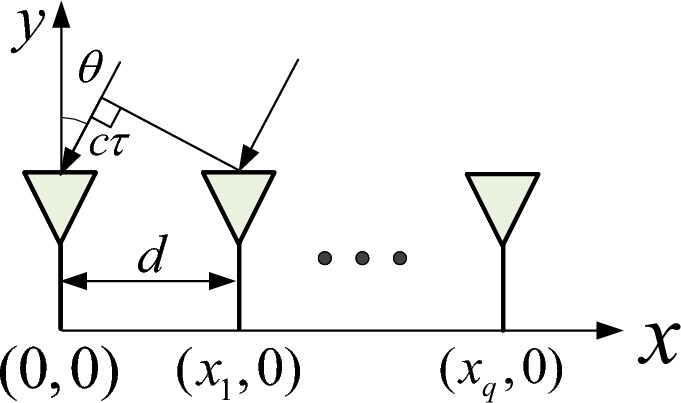
Signal reception model.

We assume $P$ narrowband signals are incident on the antenna array of the overlapping array structure, with the coordinate system set as in Fig. 2. The virtual arrays are positioned along the $x$-axis, and the array element at position $(x_q, 0)$ has spacing $d$, where $q = 0, 1, 2, \dots , Q-1$. The signal arrives in the $xOy$-plane, with the angle between the $i$-th signal’s direction and the $y$-axis as $\theta _i$, so the incident angles are $\theta _1, \theta _2, \dots , \theta _P$. The incident signal is a far-field incoherent narrowband signal. The signal vector received by the array from $P$ sources at a given time is of the form:

6 $$\begin{aligned} X(t) = \textbf{A}(\theta ) S(t) + n(t), \end{aligned}$$

where $S(t)$ is the $P \times 1$-dimensional spatial signal vector; $n(t)$ is the $Q \times 1$-dimensional noise data vector; $\textbf{A}(\theta )$ is the $Q \times P$-dimensional array manifold matrix; and $\textbf{A}(\theta ) = [a(\theta _1), a(\theta _2), \dots , a(\theta _P)]$ is the $Q \times 1$-dimensional steering vector, defined as:

7 $$\begin{aligned} a(\theta _p) = \left[ \exp (-j \varphi _{1p}), \exp (-j \varphi _{2p}), \dots , \exp (-j \varphi _{Qp}) \right] ^T, \end{aligned}$$

When there are overlapping elements in the virtual array, $a(\theta _p)$ can be expressed as:

8 $$\begin{aligned} a(\theta _p) = \left[ 1, e^{j \varphi _{2p}}, e^{j \varphi _{3p}}, e^{j \varphi _{4p}}, e^{j \varphi _{5p}}, e^{j \varphi _{6p}}, \underbrace{e^{j \varphi _{7p}}, e^{j \varphi _{8p}}}, e^{j \varphi _{9p}}, e^{j \varphi _{10p}}, e^{j \varphi _{11p}}, e^{j \varphi _{12p}}, e^{j \varphi _{13p}}, e^{j \varphi _{14p}} \right] ^T \end{aligned}$$

Performing covariance calculation on Eq. (6), the covariance matrix of the MIMO array is expressed as:

9 $$\begin{aligned} R_X&= E \left[ X(t) X(t)^H \right] \nonumber \\&= E \left[ \left( A S(t) + N(t) \right) \left( A S(t) + N(t) \right) ^H \right] \nonumber \\&= A R_S A^H + R_N, \end{aligned}$$

where $R_S$ is the signal autocorrelation matrix and $R_N$ is the noise autocorrelation matrix. Assume that the noise is Gaussian white noise and the power is $\sigma ^2$. That is to say $R_N = \sigma ^2 I$, and $I$ is a $Q \times Q$ dimensional unit matrix. $R_X$ can be expressed as:

10 $$\begin{aligned} \begin{aligned} R_x&= A R_s A^H + \sigma ^2 I \\&= \begin{bmatrix} \sum _{p=1}^{P} r_{sp} & \sum _{p=1}^{P} r_{sp} e^{-j \omega _0 (\tau _{1p} - \tau _{2p})} & \cdots & \sum _{p=1}^{P} r_{sp} e^{-j \omega _0 (\tau _{1p} - \tau _{Qp})} \\ \sum _{p=1}^{P} r_{sp} e^{-j \omega _0 (\tau _{2p} - \tau _{1p})} & \sum _{p=1}^{P} r_{sp} & \cdots & \sum _{p=1}^{P} r_{sp} e^{-j \omega _0 (\tau _{2p} - \tau _{Qp})} \\ \vdots & \vdots & \ddots & \vdots \\ \sum _{p=1}^{P} r_{sp} e^{-j \omega _0 (\tau _{Qp} - \tau _{1p})} & \sum _{p=1}^{P} r_{sp} e^{-j \omega _0 (\tau _{Qp} - \tau _{2p})} & \cdots & \sum _{p=1}^{P} r_{sp} \end{bmatrix} + \sigma ^2 I \end{aligned} \end{aligned}$$

where $r_{sp} = E \left\{ S_p(t) S_p^H(t) \right\}$; $S_p(t)$ is the $p$-th element of the $P \times 1$ dimensional spatial signal vector $S(t)$.

Due to unavoidable manufacturing and assembly errors, there exists a discrepancy between the actual and theoretical positions of the array elements. From Eq. (10), we can see that the properties of $R$ are determined only by the difference in the wavelength delay of the exponential term. Taking the origin as the reference point, $\tau _{gp}$ represents the time delay caused by the path difference of the $q$-th array element relative to the reference element. Based on the geometric relationship in Fig. 2, $\tau _{qp}$ can be expressed as:

11 $$\begin{aligned} \tau _{qp} = \frac{1}{c} (x_q \sin \theta _p) \end{aligned}$$

When there is an array position error, the position errors of the $n$-th array element in the $X$-axis and $Y$-axis directions are respectively recorded as $\Delta x_q$ and $\Delta y_q$. Then the actual position of the array element can be expressed as $(x_q + \Delta x_q, y_q + \Delta y_q)$. At this time, the delay relationship can be expressed as:

12 $$\begin{aligned} \tau '_{qp} = \frac{1}{c} \left( (x_q + \Delta x_q) \sin \theta _p + \Delta y_q \cos \theta _p \right) \end{aligned}$$

Therefore, the delay error $\Delta \tau _{qp}$ caused by the array element position error can be expressed as:

13 $$\begin{aligned} \begin{aligned} \Delta \tau _{qp}&= \tau _{qp} - \tau '_{qp} \\&= \frac{1}{c} \left( \Delta x_q \sin \theta _p + \Delta y_q \cos \theta _p \right) \end{aligned} \end{aligned}$$

Since $\varphi _d = \omega _0 \tau$, where $\omega _0 = \frac{2 \pi }{\lambda }$ is the signal angular frequency. We refer to the position errors $\Delta x$ and $\Delta y$ in the $X$-axis and $Y$-axis directions collectively as $\Delta d$. When there is an array element position error, $\varphi '_d$ is expressed as:

14 $$\begin{aligned} \begin{aligned} \varphi '_d&= \frac{2 \pi (d + \Delta d) \sin \theta }{\lambda } \\&= \frac{2 \pi d \sin \theta }{\lambda } + \frac{2 \pi \Delta d \sin \theta }{\lambda } \\&= \varphi _d + \varphi _{\text {ref}} \end{aligned} \end{aligned}$$

From Eq. (10), we can see that when there is no array position error, the elements on the diagonal of the covariance matrix $R_x$ and the elements on each diagonal line parallel to the diagonal are equal.That is to say the covariance matrix has the Toeplitz property. Combined with Eq. (14), we know that when an array position error exists, the exponential delay difference includes a term related to the position error. At this time, the Toeplitz structure of the covariance matrix is disrupted in the ideal case, which directly affects the DOA estimation performance of MUSIC.

## Methods

In this section, we propose an array element position error correction method that combines active correction and self-correction to achieve MIMO array element position error correction and improve the angle estimation accuracy of the MUSIC algorithm. First, we introduce an array element position error compensation method based on overlapping reference array elements. We use an external target with a known angle to correct the array element position error. This method is suitable for correcting the array element position error caused by antenna process errors in a laboratory environment. Secondly, we combine the overlapping reference array element method with the matrix reconstruction method and propose an array element position error correction algorithm based on matrix reconstruction. This method is suitable for the influence of system noise and array element disturbance on the angle estimation algorithm during operation.This makes up for the difficulty in obtaining external correction sources during actual operation.

### Overlapping reference array element position error correction algorithm

From Eq. (5), we can see that the first pair of overlapping elements is formed at the 7th virtual element position in the antenna array, and the second pair of overlapping elements is formed at the 8th virtual element position. We define the phase difference between the two pairs of overlapping elements as $\varphi _{O1}$ and $\varphi _{O2}$. Then, the phase difference between the first and second pairs of overlapping elements is expressed as:

15 $$\begin{aligned} {\left\{ \begin{array}{ll} \varphi _{O1} = \varphi _7 - \varphi _7' = \varphi _d + \varphi _{\text {ref}1} \\ \varphi _{O2} = \varphi _8 - \varphi _8' = \varphi _d + \varphi _{\text {ref}2} \end{array}\right. } \end{aligned}$$

where $\varphi _v$ is the phase difference generated by the target motion.

Assuming the position errors of two pairs of overlapping array elements are $\Delta d_1$ and $\Delta d_2$ respectively, the relationship between the phase change components $\varphi _{\text {ref}1}$ and $\varphi _{\text {ref}2}$ caused by the position error and the array element position error can be expressed as:

16 $$\begin{aligned} {\left\{ \begin{array}{ll} \varphi _{O1i} = \varphi _{\text {ref}1} = \frac{2 \pi \Delta d_1 \sin \theta _i}{\lambda } \\ \varphi _{O2i} = \varphi _{\text {ref}2} = \frac{2 \pi \Delta d_2 \sin \theta _i}{\lambda } \end{array}\right. } \end{aligned}$$

We calibrate the radar using strong reflectors, such as corner reflectors, to determine the compensation coefficients, thereby eliminating the effect of Doppler frequency deviation caused by target motion on angle estimation. We measure multiple stationary target data, collect radar echo data at different angles $\theta _i (i = 1, 2, \dots )$, perform distance and velocity FFT, and then extract the phase matrix of all virtual array elements to form the array manifold matrix $\hat{A}(\theta )$.

When the target is stationary, the phase difference $\varphi _v$ introduced by the target motion is 0. At this time, only the phase error $\varphi _{\text {ref}}$ caused by the array element position error will affect the angle estimation. By performing quick sampling of multiple stationary targets, and since the target angle is known, the position errors $\Delta d_1$ and $\Delta d_2$ between multiple array elements can be calculated based on Eq. (16). The average value of these errors is computed, and the average position errors between array elements are denoted as $\overline{\Delta d_1}$ and $\overline{\Delta d_2}$. At the same time, based on Eq. (16), the phase differences $\overline{\varphi }_{O1}$ and $\overline{\varphi }_{O2}$ between array elements can be obtained. The phase differences $\overline{\varphi }_{O1}$ and $\overline{\varphi }_{O2}$ are then compensated into the array manifold matrix $\textbf{A}(\theta )$ at the positions of the overlapping elements, and the compensation results are represented as:

17 $$\begin{aligned} {\left\{ \begin{array}{ll} \hat{a}_7(\theta _i)_{\text {re1}} = \hat{a}_7(\theta _i) \exp (-j \overline{\varphi }_{O1i}) \\ \hat{a}_8(\theta _i)_{\text {re2}} = \hat{a}_8(\theta _i) \exp (-j \overline{\varphi }_{O2i}) \end{array}\right. } \end{aligned}$$

We use the steering vector matrices of the two pairs of overlapping array elements after phase compensation to reconstruct the array manifold matrix $\hat{A}(\theta )$. Considering that the phase difference between adjacent virtual array elements is $\varphi _d = 2 \pi d \sin \theta / \lambda$, we use the overlapping array elements 7 and 8 as reference array elements and construct the array element phase error matrix $\Gamma$ according to Eq. (5):

18 $$\begin{aligned} {\left\{ \begin{array}{ll} r_{qi}^L = \varphi _q - \left( \varphi _7 - \frac{2 \pi (7 - q) d \sin \theta _i}{\lambda } \right) , & q = 1, 2, \dots , 6 \\[10pt] r_{qi}^R = \varphi _q + \left( \varphi _8 + \frac{2 \pi (q - 8) d \sin \theta _i}{\lambda } \right) , & q = 9, 10, \dots , Q \end{array}\right. } \end{aligned}$$

where $r_{qi}^L$ and $r_{qi}^R$ are the phase errors of array elements 1-6 and 9-14. The MIMO array phase error matrix can be expressed as:

19 $$\begin{aligned} \Gamma (r_{qi}) = \begin{bmatrix} e^{-j r_{1i}}, e^{-j r_{2i}}, \dots , e^{-j r_{6i}}, 1, 1, e^{j r_{9i}}, \dots , e^{j r_{Qi}} \end{bmatrix} \end{aligned}$$

We use the array element phase error matrix $\Gamma (r_{qi})$ to compensate the array steering vector $\hat{a}(\theta _i)$. The compensation result is expressed as:

20 $$\begin{aligned} \hat{a}(\theta _i)_{\text {ref}} = \hat{a}(\theta _i) \Gamma (r_{qi}) \end{aligned}$$

### Array element position error correction method based on matrix reconstruction

In this subsection, the overlapping reference array element position error correction method is used to compensate for the array phase error. However, in the actual radar operation process, there is a certain amount of system noise and array element disturbance, which causes array errors. Therefore, the matrix reconstruction method is introduced to reconstruct the covariance matrix in the MUSIC angle estimation method with a low rank, restore its Toeplitz structure, and thus improve the radar angle estimation accuracy.

We construct the array manifold matrix $\hat{A}(\theta _i)_{\text {re}}$ based on the phase-compensated steering vector and use the MUSIC algorithm to estimate the target angle. Considering that the number of samples of received data in practical applications is limited, the maximum likelihood estimate $\tilde{R}$ is generally used to approximate the covariance matrix $R$. The expression of $\tilde{R}$ is:

21 $$\begin{aligned} \tilde{R} = \frac{1}{L} \sum _{i=1}^{L} X X^H \end{aligned}$$

where $L$ is the number of snapshots.

When $L$ approaches $+\infty$, the error between $\tilde{R}$ and $R$ approaches 0, so the performance of the MUSIC algorithm will be reduced under the influence of a low snapshot number and system noise. We use the array manifold matrix after overlapping reference element compensation to construct the covariance matrix and first perform Toeplitz preprocessing on it. The preprocessing process is actually to take the average of the elements on the diagonal and the elements on each diagonal. Since the covariance matrix is a Hermitian matrix, only the upper triangular elements in the covariance matrix need to be processed. The processing process can be summarized as follows:

22 $$\begin{aligned} \tilde{R}_T = {\left\{ \begin{array}{ll} \tilde{r}_T(-q) = \frac{1}{Q - q} \sum _{i=1}^{Q - q} \tilde{r}_{i(i+q)}, & 0 \le q \le Q \\[10pt] \tilde{r}_T(q) = \tilde{r}_T^*(-q) \end{array}\right. } \end{aligned}$$

where $Q$ is the number of array elements; $(\cdot )^*$ represents complex conjugation; $\tilde{r}_T(-q)$ is the value of the element on the $q$-th diagonal line parallel to the main diagonal and above the main diagonal in $\tilde{R}_T$; and $\tilde{r}_{ij}$ represents the element in the $i$-th row and $j$-th column of $\tilde{R}$.

By performing eigenvalue decomposition on $\tilde{R}_T$, we extract the small eigenvalue associated with the subspace of the signal direction vector $\tilde{a}(\theta )$, and this eigenvalue can be used to form the noise subspace $\tilde{U}_N$. The MUSIC algorithm can then express the corresponding signal power as:

23 $$\begin{aligned} P_{\text {MUSIC}} = \frac{1}{{a}^H(\theta )\tilde{U}_N(\tilde{U}_N)^H {a}(\theta )} \end{aligned}$$

Under ideal conditions, the direction vector ${a}(\theta )$ is aligned with the coordinate space $\tilde{U}_N$. Therefore, in Eq. (23), the corresponding weight should be 0. However, in practical applications, due to system noise and errors, the system experiences phase shifts from its nominal value. By performing peak estimation, the estimated peak position can represent the direction of the incoming signal. However, when system noise and errors exist, the MUSIC algorithm may fail to properly reject the erroneous peaks under noisy conditions. This can be improved by using the Toepitz method, which effectively reduces such errors. Therefore, using only the Toepitz method to recover the array configuration still affects DOA estimation, especially when array elements are misaligned or heavily influenced by system noise. In such cases, the method might not fully recover the configuration, and the error limits the accuracy of angle estimation.

Assuming that the background noise is white noise, the covariance matrix of the received data usually exhibits a low-rank property due to the sparseness of the source. However, in actual traffic scenarios, the background noise is usually not pure white noise, and the environmental noise often has certain correlations or other complex characteristics, which will destroy the low-rank property of the covariance matrix. Therefore, in order to effectively suppress the interference of noise on the signal characteristics, it is necessary to construct a low-rank matrix close to the ideal covariance matrix and use this matrix to restore the key characteristic factors in the signal. Especially after Toeplitz preprocessing, the characteristics of the low-rank matrix are more significant, thereby effectively retaining the main characteristic factors in the signal space.

Considering that the nuclear norm (trace norm) is a convex relaxation function of the matrix rank, we perform a low-rank approximation on $\tilde{R}_T$ by solving the optimal solution of the nuclear norm optimization problem.

24 $$\begin{aligned} \begin{aligned} \min _{R_0} \quad&\Vert R_0 \Vert _* \\ \text {subject to} \quad&\Vert \tilde{R}_T - R_0 \Vert _F \le \epsilon \end{aligned} \end{aligned}$$

where $\Vert \cdot \Vert _*$ represents the trace norm; $\Vert \cdot \Vert _F$ represents the Frobenius norm; $\epsilon$ represents the tolerance to noise, which is generally determined by the noise level. Noise tolerance is expressed as:

25 $$\begin{aligned} \epsilon = Q \times \sigma _n^2 \end{aligned}$$

In order to reduce the amount of calculation and enhance robustness, the Toeplitz property of the covariance matrix is used. We let $R_0 = toep(u)$, where $u$ is the row vector composed of the first row elements of $R_0$, so Eq. (24) can be transformed into:

26 $$\begin{aligned} \begin{aligned} \min _{u} \quad&\Vert toep(u) \Vert _* \\ \text {subject to} \quad&\Vert \tilde{R}_T - toep(u) \Vert _F \le \epsilon \end{aligned} \end{aligned}$$

**Fig. 3:**
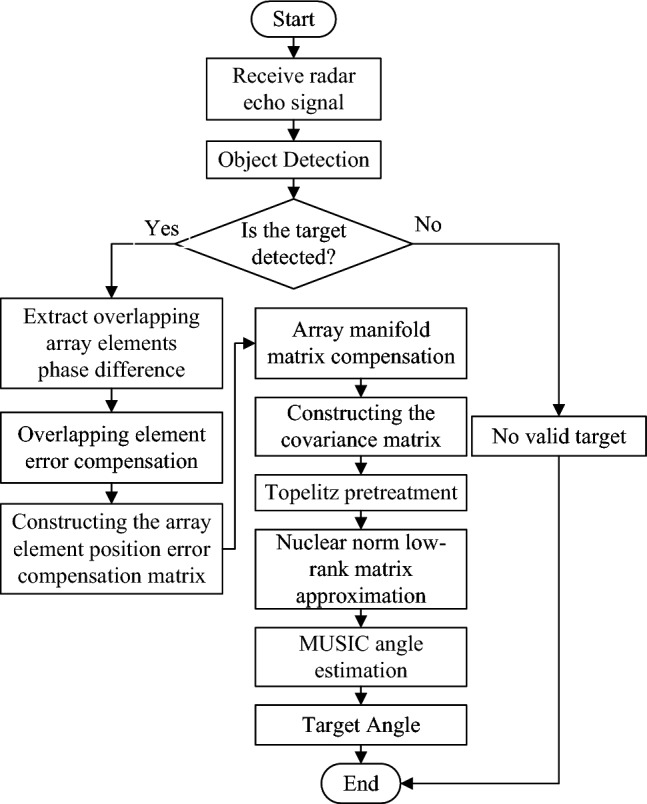
Array element position error correction and angle estimation algorithm flow chart.

The optimized variable is reduced from a matrix to a single vector, which reduces the computational complexity. We further relax Eq. (26), and the optimized equation is expressed as:

27 $$\begin{aligned} \min _{u} \quad \frac{1}{2} \Vert \tilde{R}_T - toep(u) \Vert _F^2 + \mu \Vert toep(u) \Vert _* \le \epsilon \end{aligned}$$

where $\mu$ is a regularization parameter, and $\mu > 0$, which is used to balance the influence of the first and second terms on the optimization results. We use the convex optimization toolbox to obtain the optimal solution, denoted as $R^*$, which is the covariance matrix after matrix reconstruction. We perform eigen decomposition on $R^*$ and finally use the MUSIC algorithm to obtain the target angle estimate. The spectral function of the MUSIC algorithm is expressed as:

28 $$\begin{aligned} P_{MUSIC}(\theta ) = \frac{1}{a^H(\theta ) U_N^* (U_N^*)^H a(\theta )} \end{aligned}$$

where $U_N^*$ is the noise subspace eigenvector matrix corresponding to the $Q - P$ small eigenvalues after eigen decomposition of $R^*$. The specific process of array element position error correction and angle estimation algorithm is shown in Fig. 3.

## Simulation and experiment analysis

In this section, we verify the effectiveness of our method through computer numerical simulation experiments and actual traffic scenario tests.

### Simulation analysis

In this subsection, we verify the effectiveness of our algorithm through a series of computer numerical simulation experiments. We designed two simulation scenarios, a single target scenario and a multi-target scenario. We set the array element position error d1 to obey a uniform distribution, and further verify the effectiveness of our method by comparing the angle estimation results of our method with the reference array element compensation method and the traditional Toeplitz correction method, and analyzing the Monte Carlo experimental results.

#### Simulation setup

Our radar antenna array uses 4-TX and 4-RX to form 14 virtual array elements, including 2 pairs of overlapping array elements. The array element spacing is set to $d = 1.5\lambda$. The array layout structure is shown in Fig 1. The radar signal waveform uses a sawtooth frequency modulated continuous wave. The transmitting antenna transmits signals in the order of TX1, TX4, TX2, and TX3. The receiving antenna receives the echo signal at the same time and processes the echo signal using our algorithm. The millimeter wave radar parameters are shown in Table 1, and the simulated target parameters are shown in Table 2.

**Table 1:** Millimeter wave radar parameters.

Radar parameters	Simulation values	Measured values
Carrier frequency $f_0 (GHz)$	60	60
Modulation period $T (\mu s)$	45	45
FM-bandwidth *B*(*MHz*)	200	200
Number of chirps $N_d$	128	128
Distance resolution (m)	0.75	0.75
Speed resolution (m/s)	0.108	0.108
Angular resolution ($^{\circ }$)	2.7	2.7

**Table 2:** Simulation target parameters.

Target parameters	Target distance (m)	Target speed (m/s)	Target Angle (°)
Target 1	18	20	-6.5
Target 2	55	0	1.5
Target 3	80	5	20

For the influence of target angle bias on the position error of array elements, based on Eq. (16), it can be derived that$\Delta d = \frac{\lambda }{2 \pi } \sin (\theta )$. If the target angle $\theta$ has a small bias $\Delta \theta$, the corresponding error in $\Delta d$ is approximately:

29 $$\begin{aligned} \frac{\Delta (\Delta d)}{\Delta d} \approx \frac{\cos (\theta )}{\sin (\theta )} \Delta \theta \end{aligned}$$

Thus, as $\theta$ approaches 0$^{\circ }$, the sensitivity increases, and as $\theta$ moves further from 0$^{\circ }$, the sensitivity decreases, which can be verified through practical experiments by inserting angle biases of $\pm 0.1^\circ$, $\pm 0.2^\circ$, and $\pm 0.5^\circ$, as shown in Table 3.

**Table 3:** Relationship between target angle bias ($\Delta \theta$) and relative position error in $\Delta d$.

Angle Bias ($\Delta \theta$)	Relative Error in $\Delta d$ (%)
$\pm 0.1^\circ$	0.19%
$\pm 0.2^\circ$	0.38%
$\pm 0.5^\circ$	0.95%

The results show that in practical applications, when the reference target has an angle bias, the influence on the final position accuracy is negligible.

For the selection of the normalization factor $\mu$, we set the received signal’s SNR to 0 dB, with the sampling rate set to 500. The normalization factor $\mu$ is then determined using the MUSIC algorithm. After processing the signal, a group of MUSIC angle measurements is carried out to find the optimal performance corresponding to the normalization factor $\mu$. The final experiments are performed based on the pre-measured group of experimental methods to confirm the value of the normalization factor.

For the selection of the signal-to-noise ratio (SNR), we calculate the corresponding results based on different signal-to-noise ratios and sampling numbers, as shown in Table 4 and Table 5. The results indicate that the selection of SNR and the sampling rate affects the corresponding errors as shown in Table 4 and Table 5.

**Table 4:** The relationship between SNR and $\epsilon$.

SNR (dB)	Relative Error in $\epsilon$ (%)
-8	15
-6	14
-4	13
-2	12
0	10
2	10
...	...
16	10

**Table 5:** The relationship between sampling rate and $\epsilon$.

Sampling Rate	Relative Error in $\epsilon$ (%)
200	12
300	11.5
400	11
...	...
900	8.5
1000	8

#### Single-target and multi-target scenario verification simulation results

**Fig. 4:**
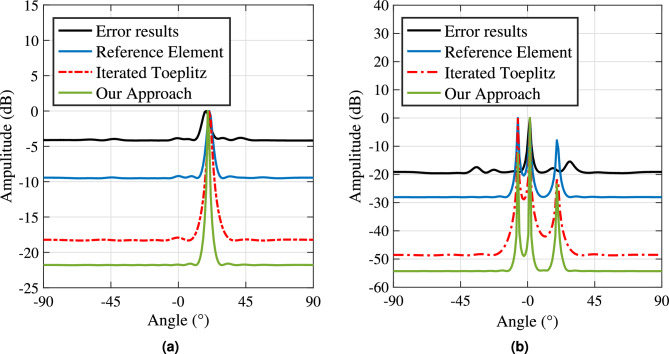
Angle estimation results. (**a**) Single target angle estimation comparison results. (**b**) Multiple target angle estimation comparison results.

We select target 3 as the verification target for the single target scene, and target 1, target 2, and target 3 for the multi-target scene. Our method is compared with the angle estimation results when position error exists, the reference array method^8^, and the iterated Toeplitz method^18^. In the single target scenario, $\mu = 0.08$, and in the multi-target scenario, $\mu = 0.15$. The simulation results are shown in Fig. 4.

Figure 4 presents the simulation results for angle estimation. To maintain a consistent distribution probability for the array element position error within a specified range, this error is modeled using a uniform distribution. In Fig. 4a, we illustrate the results for a single-target scenario, while Fig. 4 b depicts the outcomes for a multi-target scenario.

The figures clearly show that the presence of array element position errors significantly degrades the performance of the MUSIC algorithm. The spectrum peaks become less pronounced, leading to deviations in angle estimation results. When comparing our method to the reference array element method and the iterative Toeplitz method, it is evident that our approach yields sharper peaks in the MUSIC spectrum and smaller angle estimation errors in both single-target and multi-target scenarios. This further highlights the advantages of our method in enhancing angle estimation performance for the MUSIC algorithm. The corresponding angle estimation result data is presented in Table 6.

#### Monte Carlo simulation experiment results

**Fig. 5:**
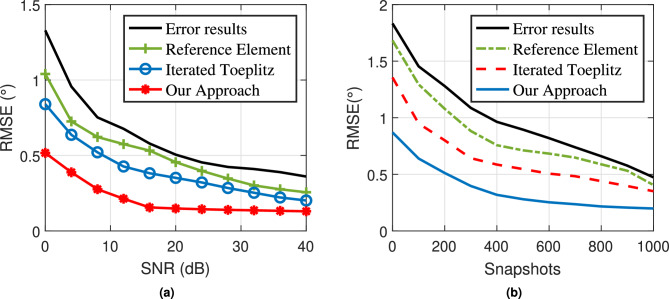
Error compensation algorithm performance. (**a**) SNR-RMSE comparison results. (**b**) Snapshot number-RMSE comparison results.

To further assess the effectiveness of our proposed algorithm, we performed Monte Carlo experiments to analyze the relationships between the signal-to-noise ratio (SNR) and root mean square error (RMSE), as well as between the number of snapshots and RMSE. For the SNR-RMSE experiment, we fixed the number of snapshots at 200, and for the snapshot number-RMSE experiment, we set the SNR to 0 dB. The results are presented in Fig. 5.

**Table 6:** Simulation data results.

Target parameters	Uncorrected Angle($^{\circ }$)	Reference Element($^{\circ }$)	Iterated Toeplitz($^{\circ }$)	Our Approach($^{\circ }$)
Target 1	-9.55	-6.75	-6.82	-6.54
Target 2	1.52	1.52	1.54	1.51
Target 3	18.60	19.64	20.65	19.86

Our algorithm was compared with both the reference array element method and the iterative Toeplitz algorithm. The reference array element method utilizes the first array element as a reference, adjusting for array phase based on the phase difference across two frequency modulation cycles of this reference element. However, this method overlooks phase deviations in the reference element itself, resulting in inconsistent correction of array element position errors. The iterative Toeplitz algorithm partially alleviates the impact of array element position error on angle estimation, but it incurs a high computational cost due to iterative processing, and its performance gains are limited, especially under low snapshot conditions.In contrast, the algorithm proposed in this paper addresses the reference array element position error, reduces the computational burden in Toeplitz matrix reconstruction, and decreases system noise. The RMSE achieved by our algorithm remains stable at around 0.2$^{\circ }$. Compared with the other two algorithms, our approach shows clear advantages in both SNR and snapshot number, making it more robust and efficient under various experimental conditions.

### Experiment analysis

In this section, we use actual traffic roads as experimental scenarios and conduct experimental tests in actual scenarios to verify the adaptability of our method in actual scenarios.

#### Experiment setup

The vehicle target echo data collected by FMCW radar in the actual measurement scene was used. The radar hardware was developed by our R&D team and is composed of Calterah’s CAL60S244 chip and Xilinx’s ZYNQ-7020 chip. The antenna array layout is shown in Fig. 1, which includes 4-TX and 4-RX antennas, where M=4 and N=4. The raw data was collected for PC experiments.

**Fig. 6:**
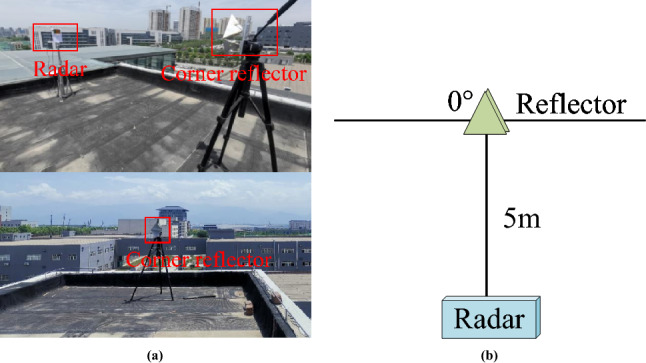
Static experimental scene. (**a**) Radar and corner reflector. (**b**) Static scene coordinate system structure.

We set up a stationary target scene and used a corner reflector to replace the stationary vehicle target. The experimental scene layout is shown in Fig. 6. The straight-line distance between the radar and the corner reflector is 5m, and the azimuth angle of the corner reflector relative to the radar is 0 degrees.

**Fig. 7:**
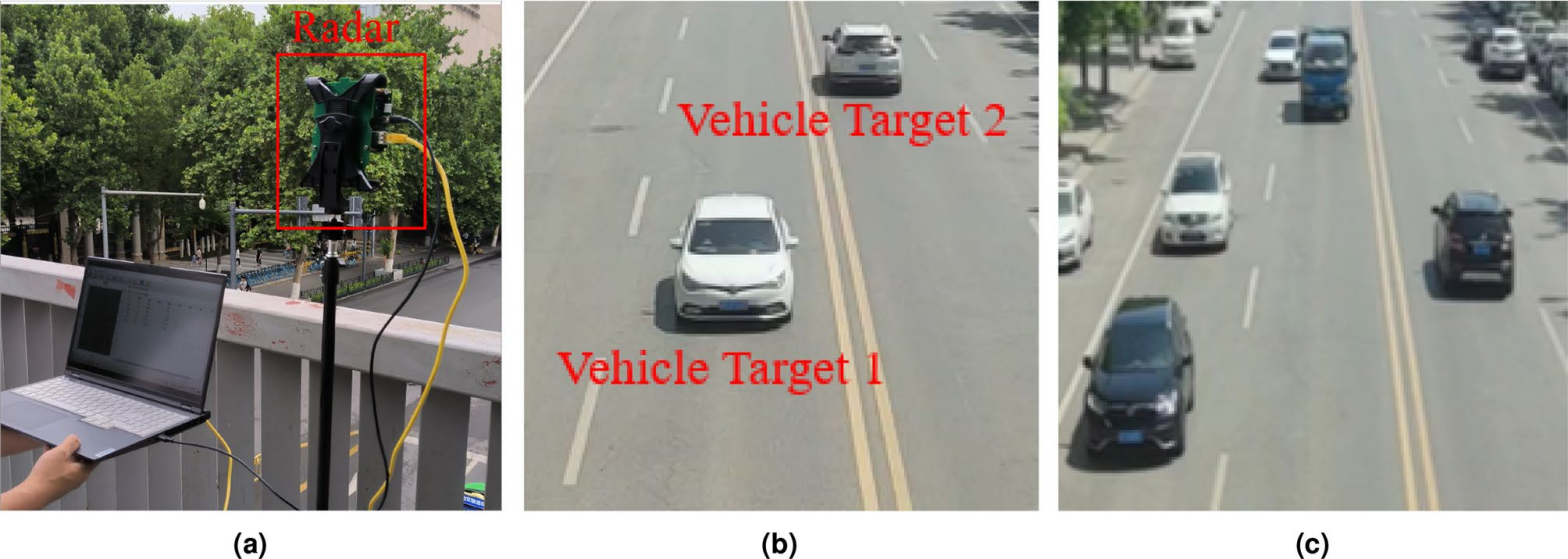
Actual target scene. (**a**) radar hardware equipment. (**b**) Scene with 2 vehicle targets. (**c**) Scene with 5 vehicle targets.

We set the moving target scene to the actual traffic road scene. The actual measurement scene is a two-way lane of an urban road. The radar is placed above the overpass and faces the lane to collect data. The actual scene is as shown in the Fig. 7.

The detection target is a moving vehicle target. The millimeter-wave radar parameters are shown in Table 1, We tested the 2-vehicle target and 5-vehicle target experimental scenarios respectively, and analyzed the 2-vehicle target scenario. The 2-vehicle target parameters are shown in Table 7.

**Table 7:** Actual target parameters.

Target parameters	Target 1	Target 2
Range (m)	31	46
Velocity (m/s)	14.2	11.9
Angle ($^{\circ }$)	-7.5	2.5
Measured range (m)	31.13	45.77
Measured velocity (m/s)	14.31	11.92
Uncompensated angle ($^{\circ }$)	-29.66	-18.60
Measured angle ($^{\circ }$)	-7.63	2.64

#### Experiment results

**Fig. 8:**
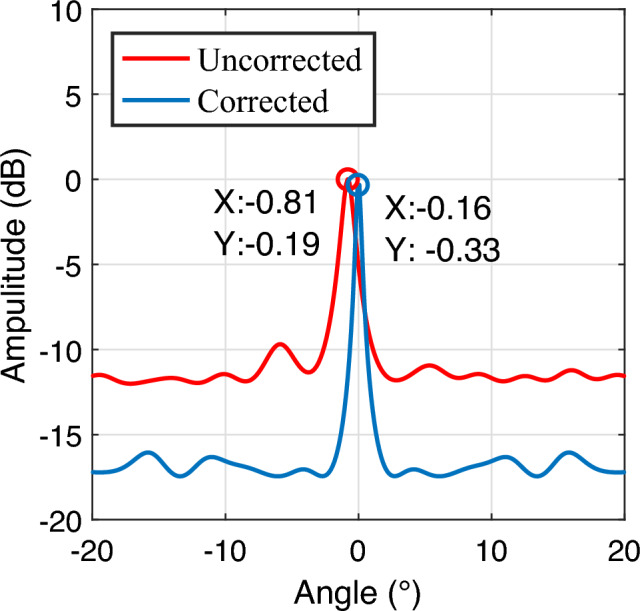
Results of stationary target experiments.

We use Matlab2023b software to process the echo data collected from the actual road, and estimate the angle of the target after 2D-FFT processing. Fig. 8 shows the angle estimation result of the stationary target. The result shows that the angle estimation error of the target facing the radar is 0.16 degrees. The target angle estimation is accurate and meets the actual angle measurement conditions.

**Fig. 9:**
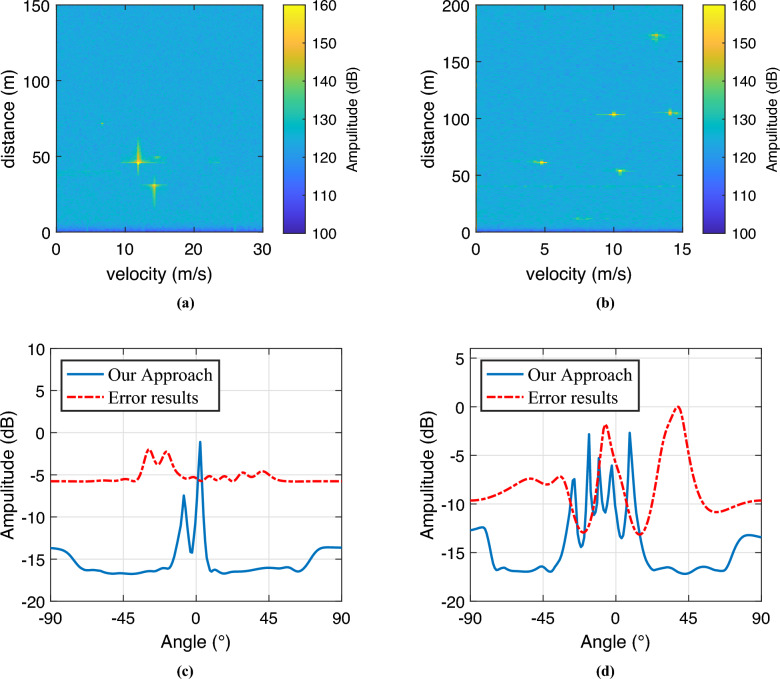
Angle estimation experimental results. (**a**) 2D spectrogram of 2 targets. (**b**) 2D spectrogram of 5 targets. (**c**) Angle estimation results of 2 targets. (**d**) Angle estimation results of 2 targets.

Figure 9 shows the results of multi-target detection, where Fig. 9a and Fig. 9b are the 2D-FFT spectrum results of vehicles, and Fig. 9c and Fig. 9d are the angle estimation results of vehicles. The maximum peak point cloud of the two-vehicle target is extracted for MUSIC angle estimation, and the MUSIC angle estimation results are analyzed. Figure 9bcompares the MUSIC angle estimation results before and after error correction. The two peaks in the figure represent two targets. When not corrected, the angle estimation results have a large error with the actual value. After correction, the angle estimation results of target 1 and target 2 are -7.63$^{\circ }$ and 2.64$^{\circ }$, respectively, which are basically consistent with the actual angles.

To verify the feasibility of the algorithm’s extension in practical systems, we conducted a computational overhead test. The nuclear norm optimization time was tested on a laptop equipped with an Intel Core i5-12500 processor (3.0 GHz, 6 cores, 8 threads) and 16 GB of RAM. For a 14$\times$14-sized virtual array covariance matrix, nuclear norm optimization reconstruction was performed, with the average processing time per optimization being 6.4 milliseconds. Considering that the frame rate of millimeter-wave traffic radar systems typically ranges from 20 to 50 milliseconds per frame, our method can run in real time under these hardware conditions, with a time usage ratio of approximately 12%-32%, thus meeting the latency requirements for real-time traffic monitoring applications.

Experimental results show that in actual traffic scenarios, the MIMO radar array element position error correction method based on overlapping reference array element matrix reconstruction has a good correction effect on the array element position error, which can effectively reduce the influence of the array element position error on the angle estimation, meet the needs of actual traffic applications, and verify the feasibility of the algorithm.

## Conclusion

This paper studies the MIMO array element position error correction method. Aiming at the problem that the array element position error caused by antenna manufacturing process errors and array element disturbances in the operating state reduces the performance of the angle estimation algorithm, a reconstruction of the overlapping reference element matrix is proposed. The array element position error correction method uses a combination of external correction sources and self-correction to achieve array element position error correction, and uses the MUSIC algorithm to achieve target angle estimation. Its effectiveness is verified through simulation and experiment. Experimental results show that compared with traditional methods, this method effectively solves the angle estimation error caused by array element position error, and the estimation accuracy is stable within 0.2$^{\circ }$. In addition, by utilizing Toeplitz matrix reconstruction, the system noise is significantly reduced, enabling the radar to achieve high detection accuracy under low signal-to-noise ratio and limited snapshots. This method also has good angle estimation performance when the target distance is relatively close, and has broad application potential in complex traffic scenes, autonomous driving and other related fields. Since our method is an unsupervised optimization approach, it can be combined with neural network optimization methods in the future, which will be the next research direction.

## Data Availability

The data involve the privacy and confidentiality agreements of the research subjects, and some of the data are shared under commercial collaboration agreements. Therefore, we are unable to provide the data separately. However, upon reasonable request, the data can be obtained from the corresponding author.
